# The Role of Ventriculocisternostomy in the Management of Hydrocephalus in Mali and the Democratic Republic of the Congo

**DOI:** 10.7759/cureus.59189

**Published:** 2024-04-28

**Authors:** Tshiunza Mpoyi Chérubin, Kabongo Augustin, Ntalaja Jeff, Mirenge Goert, Metre Guelord, Manuel de Jesus Encarnacion Ramirez, Beltchika Antoine, Maoneo Israël, Mukuetala Pierre, Kisubi Michel, Punga Ziko, Ketani Teddy, Ouhdiri Yassad, Medhi Hakou, Ntsambi Glennie, Nicola Montemurro

**Affiliations:** 1 Neurosurgery, Centre Hospitalier Initiative Plus, Kinshasa, COD; 2 Neurosurgery, Université de Mbujimayi, Mbuji Mayi, COD; 3 Neurological Surgery, Peoples' Friendship University of Russia, Moscow, RUS; 4 Neurosurgery, Université de Kinshasa, Kinshasa, COD; 5 Neurosurgery, Hôpital des Spécialités de Rabat, Rabat, MAR; 6 Neurosurgery, Azienda Ospedaliero Universitaria Pisana, Pisa, ITA

**Keywords:** low-income countries, neurosurgery, patient outcomes, adult hydrocephalus, ventriculocisternostomy

## Abstract

Background: Hydrocephalus continues to pose significant clinical challenges in neurosurgery. The primary goal of this study is to assess the feasibility of ventriculocisternostomy (VCS) within the provincial city of Kinshasa and Mali to optimize the management of patients afflicted with hydrocephalus.

Methods: This investigation was hosted at two major urban healthcare facilities: the Initiative Plus Hospital Center, positioned in the bustling metropolis of Kinshasa, Democratic Republic of the Congo (DRC), and the Bamako Hospital, Republic of Mali. A prospective, analytical cohort study was executed from December 2022 to June 2023.

Results: In the Mali group, seven patients underwent VCS, four patients were treated with VCS and spinal surgery, and one case was treated with VCS and biopsy. Similarly, in the Kinshasa group, 25 patients underwent VCS, whereas four patients were treated with VCS and spinal surgery. The median hospital stay was eight and 10 days for the Mali and the Kinshasa groups, respectively.

Conclusion: VCS emerges as a formidable alternative for hydrocephalus management in Mali and DRC, showcasing the potential to markedly ameliorate patient outcomes, economize healthcare expenditures, and fortify the local neurosurgical capacity.

## Introduction

Hydrocephalus, characterized by the abnormal accumulation of cerebrospinal fluid (CSF) within the ventricles of the brain, continues to pose significant clinical challenges in neurosurgery. Traditionally managed with shunt systems, the condition has witnessed progressive therapeutic advances, particularly with the advent of endoscopic ventriculocisternostomy (EVC), a minimally invasive technique offering a shunt-free alternative for selected patients. This article explores the burgeoning significance of EVC, specifically in the context of the city province of Kinshasa in the Democratic Republic of the Congo (DRC), where the burden of neurosurgical disorders is profoundly influenced by regional healthcare disparities [1-3].

In recent years, scholarly discussions have increasingly highlighted the disparities in neurosurgical care across various geopolitical landscapes. The DRC, with its unique socio-economic and healthcare infrastructure, presents a microcosm where the impact of such advancements can be distinctly measured [4]. Ventriculocisternostomy (VCS), primarily in the form of endoscopic third ventriculostomy (ETV), has been shown to circumvent many of the complications associated with ventriculoperitoneal (VP) shunts, such as infections and mechanical failures [5]. The efficacy of EVC is predicated on the patient's underlying pathology, with the best outcomes reported in obstructive or "non-communicating" hydrocephalus [6]. In Kinshasa, where resource limitations amplify the logistical challenges of managing chronic conditions, EVC presents as a particularly attractive intervention.

Recent evidence from the region suggests a paradigm shift, with endoscopic techniques being increasingly favored over shunt placements [7,8]. This trend underscores the necessity for contextual analyses, as the benefits of EVC must be juxtaposed with regional expertise, equipment availability, and patient selection criteria. Furthermore, the sustainability of EVC in Kinshasa's healthcare ecosystem necessitates a comprehensive approach that incorporates training, infrastructure development, and continuous outcome monitoring.

The primary goal of this study is to assess the feasibility of VCS within the provincial city of Kinshasa to optimize the management of patients afflicted with hydrocephalus.

Specific objectives of this study are (1) to illustrate the significance and practicality of VCS in the treatment of hydrocephalus within our local healthcare framework; (2) to identify the specific clinical indications for the employment of this technique in the context of our medical practice; and (3) to evaluate the outcomes of VCS in a preliminary cohort of carefully chosen patients, thereby establishing an evidence base for its effectiveness and utility.

## Materials and methods

Study design and setting

This investigation was hosted at two major urban healthcare facilities: the Initiative Plus Hospital Center, positioned in the bustling metropolis of Kinshasa, Democratic Republic of the Congo, and the Bamako Hospital, Republic of Mali.

The study was conducted in accordance with the Declaration of Helsinki and approved by the Ethics Committee of the "Ministère de l'Enseignement Supérieur et Universitaire," University of Kinshasa, Democratic Republic of the Congo (February/2024).

A prospective, analytical cohort study was meticulously planned and executed from December 2022 to June 2023. This design was selected to enable a robust longitudinal analysis, permitting us to closely track clinical progress and capture the nuanced outcomes of VCS as a treatment modality for pediatric hydrocephalus. The study was geared toward generating a comprehensive dataset that would reflect the efficacy, safety, and practicability of VCS in a real-world clinical setting.

A scientifically rigorous approach was employed to determine the sample size. Utilizing a standardized formula, we calculated the minimum sample size required to achieve statistically significant results. The formula incorporated the following elements: n = the requisite sample size, z = the z-score corresponding to a 95% confidence level (1.96), p = the assumed prevalence rate (50%), and m = the margin of error permitted within the study (7%). This formula yielded a target sample size of 50 patients, ensuring that our study would be sufficiently powered to detect meaningful differences and trends.

Study Population

Our study population was composed of pediatric patients diagnosed with hydrocephalus and identified as suitable candidates for VCS. Inclusion spanned across the city and provinces of Kinshasa and Bamako, with patient management being consistently conducted by an adept medical team dedicated to this study. By engaging a demographically diverse cohort within two different healthcare infrastructures, our study was uniquely positioned to interrogate the broad applicability and potential disparities in VCS treatment outcomes.

Inclusion and Exclusion Criteria

To curate a representative cohort, stringent inclusion and exclusion criteria were established. We included patients from newborns up to 10 years old with a confirmed diagnosis of hydrocephalus who were referred for VCS. Consent from a parent or legal guardian was a prerequisite for inclusion in the study. Conversely, we excluded patients presenting with inactive hydrocephalus, those beyond the age of 10 years, or any who lacked the necessary consent to partake in the study.

Data Collection and Analysis

Data collection was designed to be both methodical and comprehensive, encompassing demographic information, clinical presentations, diagnostic imaging findings, therapeutic interventions, and postoperative outcomes. All collected data were meticulously logged into a secure digital database, ensuring patient confidentiality and data integrity. Advanced statistical software was employed to facilitate detailed data analysis, with findings set to inform future clinical guidelines and policy-making in the treatment of pediatric hydrocephalus.

Equipment

To ensure a standardized procedure conducive to high-quality outcomes and replicability, all VCS interventions were executed using a state-of-the-art, 2 mm diameter, 0-degree rigid Karl Storz® endoscope (Tuttlingen, Germany). This equipment represents the gold standard in neuroendoscopic equipment, providing superior optics and maneuverability crucial for the precision required in pediatric neurosurgery. The endoscope was accompanied by an ensemble of endoscopy column accessories, including a high-definition camera and its corresponding cable for visual guidance, a cable connected to a high-intensity cold light source ensuring optimal illumination while minimizing thermal injury risk, a monopolar fiber for cauterization purposes, stoma forceps, and biopsy forceps designed for meticulous tissue handling and sample collection. Additionally, a Fogarty catheter, a trocar with a mandrin, and a quadruple-port operative sheath were employed to facilitate instrument insertion and fluid management during the procedures.

Sociodemographic Variables

Comprehensive sociodemographic data were collected to control for potential confounders and to allow for detailed subgroup analysis. The variables included patient age, date of birth, gender, and marital status (of the parents, for pediatric patients), along with the profession, site of care (Initiative Plus Hospital Center in Kinshasa, and its Bamako counterpart), parental age, mode of delivery, geographical origin indicating ethnicity to account for genetic predispositions, consanguinity, and a detailed medical-surgical history of the mother, which might impact the pediatric patient's health.

Diagnostic Variables

Consultation and antenatal period: The initial patient consultations and any reports from the antenatal period were reviewed to ascertain the early detection and progression of hydrocephalus.

Imaging data: Diagnostic imaging played a pivotal role in the confirmation of hydrocephalus and its categorization into etiological subtypes. The suite of imaging modalities included transfontanellar ultrasound for its non-invasiveness and utility in initial screenings, brain CT scans complemented by the Evans ratio for quantifiable assessment of ventricular size, and MRI for high-resolution imaging of the brain's structure and the identification of potential complications or comorbid conditions.

Types of Hydrocephalus

Based on imaging and clinical assessments, hydrocephalus was categorized into communicating hydrocephalus (indicating an issue with CSF absorption) and non-communicating hydrocephalus, where CSF flow is obstructed within the ventricular system.

Therapeutic data

Medical Treatment

Prior to any surgical intervention, medical management was administered and included (1) antibiotics with analgesics to manage infection and pain or (2) antibiotics with antimalarial drugs in regions where malaria is endemic. A comprehensive regimen of antibiotics, analgesics, antimalarials, and antifungals was included when indicated by patient history and clinical judgment.

Surgical Treatment

The surgical approach encompassed VCS procedures, initially performed by an experienced visiting neurosurgeon, with subsequent surgeries conducted by our in-house team starting from the fifth VCS. VP shunts were also employed as required, based on individual patient indications.

Patient Outcomes

The study meticulously recorded postoperative complications, including infectious complications, CSF leaks at the surgical site, serous effusions, intraoperative hemorrhage, and patient mortality rates. The duration of postoperative hospitalization was tracked to evaluate the immediate recovery period and to correlate the length of stay with the incidence of postoperative complications.

## Results

From December 2022 to June 2023, 12 patients from the Mali group and 29 patients from the Kinshasa group were treated.

Age

Combining the two groups, the median age was six months, with an SD of 1.75. There was no statistically significant difference in age between the Mali and Kinshasa groups, given a conventional alpha level of 0.05.

Birth weight

The combined data yielded a median weight closer to the Kinshasa group, and the SD remained the same as Kinshasa's. The difference in birth weights between the two groups was not statistically significant.

Hospital stay

The combined median was nine days, with an SD almost as high as that for the Kinshasa group alone. The p-value of 0.098 was approaching significance, indicating that there may be a trend toward longer hospital stays for the Kinshasa group.

Fever during pregnancy and consanguinity

Fever During Pregnancy

More cases were reported in the Kinshasa group (N = 7) than in the Mali group (N = 5). However, without a total number of pregnancies for context, it is challenging to interpret the significance of these numbers.

Consanguinity

The Kinshasa group showed a higher number of cases (N = 6) compared to the Mali group (N = 3). This suggests a higher rate of blood relation between parents in the Kinshasa group. Consanguinity is a significant risk factor for many genetic disorders, which may be relevant to the clinical outcomes of interest.

Postoperative Fever

Mali group: There were five cases of postoperative fever.

Kinshasa group: There were six cases of postoperative fever.

Regarding clinical aspects, the data show no significant difference in postoperative fever incidence, which might indicate that the surgical care and infection control measures are consistently applied across groups. However, the higher reports of fever during pregnancy and consanguinity in the Kinshasa group may point to differing preoperative risk factors or genetic backgrounds that could impact patient outcomes. Table 1 shows all the details.

**Table 1:** Demographic and clinical data

Variable	Group	No.	Median (SD)	P-value
Age (months)	Mali	-	5.00 (1.07)	-
	Kinshasa	-	8.00 (2.00)	-
	Overall	-	6.00 (1.75)	0.200
Birth weight (kg)	Mali	-	3.90 (0.14)	-
	Kinshasa	-	3.48 (0.55)	-
	Overall	-	3.54 (0.55)	0.299
Hospital stay (days)	Mali	-	8.00 (2.12)	-
	Kinshasa	-	10.00 (4.68)	-
	Overall	-	9.00 (4.71)	0.098
Fever during pregnancy	Mali	5 (41.7)	-	-
	Kinshasa	7 (24.1)	-	-
	Overall	12 (29.3)	-	-
Consanguinity	Mali	3 (25)	-	-
	Kinshasa	6 (20.7)	-	-
	Overall	9 (22.0)	-	-
Postoperative fever	Mali	5 (41.7)	-	-
	Kinshasa	6 (20.7)	-	-
	Overall	11 (26.8)	-	-

Age at symptom onset

Mali Group

The median age at symptom onset was one month, with an SD of 1.00 months, suggesting that symptoms tend to present early in life, but there is variability, possibly due to different conditions within the patient group or variability in when parents notice or report symptoms.

Kinshasa Group

This group had a slightly higher median age at symptom onset of two months. The combined overall median age at symptom onset was 1.5 months, and the SD was slightly higher than the individual groups at 2.10 months. The identical p-values (0.338) for both groups and the overall population indicate there is no significant difference in the age at symptom onset between the two groups.

Sex

The sex distribution shows a male predominance (60%), which could suggest either a higher incidence of the underlying conditions among males, differences in health-seeking behavior for male and female children, or a possible referral bias.

Current weight

Mali Group

The median current weight was 5.0 kg with an SD of 1.23 kg, which, depending on the age distribution of the population, could be indicative of normal growth or possible health issues.

Kinshasa Group

There was a marked increase in the median current weight at 10.0 kg, with a considerably higher SD of 3.36 kg, suggesting not only a greater weight but also a much wider spread in the weight range, potentially pointing to a more varied age range or different nutritional statuses among patients.

The combined overall median current weight was 6.5 kg, and the SD was high at 3.30 kg, closer to the Kinshasa group, reflecting this group's influence on the overall dataset due to their higher weight variance. Age at symptom onset and current weight were the same across individual groups and overall (0.338 and 0.115, respectively), indicating that any observed differences are not statistically significant. For instance, while the differences in current weight between the two groups are not statistically significant, the Kinshasa group's median weight is double that of the Mali group, which could be clinically significant, especially considering nutritional status or growth patterns in relation to health outcomes. Additionally, the high standard deviations, especially in the Kinshasa group for current weight, could indicate a diverse patient population or a wide range of conditions affecting weight. It may also reflect different health interventions, nutrition programs, or socioeconomic factors affecting the patient cohorts in Mali and Kinshasa. Tables 2, 3 show all the details.

**Table 2:** Age at symptom onset and current weight of patients from Mali and Kinshasa

Group	Age at symptoms (months), median	Age at symptoms (months), SD	Current weight (kg), median	Current weight (kg), SD	Age at symptoms, P-value	Current weight, P-value
Mali	1.5	1.00	5.0	1.23	0.338	0.115
Kinshasa	2.0	2.17	10.0	3.36	0.338	0.115
Overall	1.5	2.10	6.5	3.30	0.338	0.115

**Table 3:** Clinical data

Variable	Group	No. (%)
Vaccination	Mali	12 (100)
	Kinshasa	28 (96.6)
Folic acid intake	Mali	12 (100)
	Kinshasa	26 (89.7)
Mode of delivery		
- Dystocia	Mali	1 (8.3)
- Eutocic	Mali	11 (91.7)
- Eutocic	Kinshasa	28 (96.6)
- Cesarean section	Kinshasa	1 (3.4)
Fontanelle bulging	Mali	6 (50.0)
	Kinshasa	14 (48.3)

Use of antibiotics

Mali

A total of 70% of patients were treated with cefotaxime. This suggests a standardized approach to managing infections or preventing infection post surgery.

Kinshasa

A similar proportion (70%) received Cefotaxime, but 30% were treated with ceftriaxone. The use of two different antibiotics might indicate a broader range of bacterial infections or a tailored approach to antibiotic resistance patterns.

Use of analgesics and anti-inflammatory drugs

Both groups used paracetamol (100%), indicating it is the standard analgesic for managing pain in these patients. In both groups, nifluril was used for 70% of patients, and Profenid for 30%, which is a common approach to managing inflammation, potentially to reduce intracranial pressure or postoperative swelling.

Type of hydrocephaly

Mali

There were three cases of tetraventricular, eight cases of atrioventricular, and one case with no dilation hydrocephaly, suggesting a predominance of triventricular hydrocephaly in this group.

Kinshasa

The presence of more triventricular cases (n = 23) compared to tetraventricular (n = 3) and no dilation hydrocephaly (n = 3) was noted. This implies a similar distribution but with a larger number of patients overall, reflecting possibly a larger sample size or a higher prevalence of the condition in this region.

Surgery performed

Mali

Most patients underwent VCS surgery, with a few requiring additional spinal surgery or biopsy, indicating varying severity and complexities of cases.

Kinshasa

**Figure 1:**
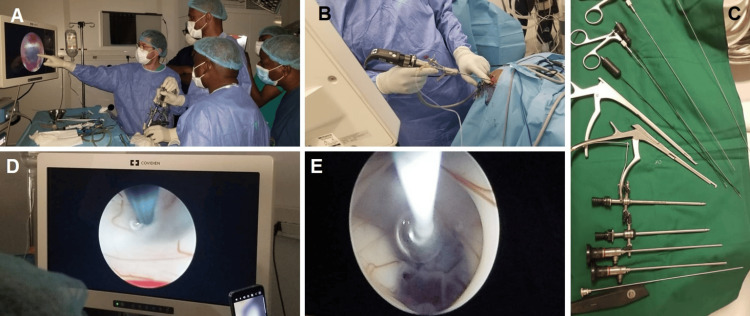
Surgical steps (A-B) Surgeons at work with an endoscope; (C) surgical instruments; (D-E) endoscopic third ventriculostomy.

A larger number of patients underwent VCS surgery, with a small portion needing additional spinal surgery, showing a similar surgical approach to Mali but on a larger scale (Figure 1).

Imaging of suspected anomalies

In the Mali group, there were specific cases like Dandy-Walker syndrome and intraventricular tumors, highlighting the necessity for detailed imaging and diagnostics in managing hydrocephaly. Table 4 shows all the details.

**Table 4:** Surgical treatment details VCS: ventriculocisternostomy.

Group	Antibiotic used	Analgesic used	Anti-inflammatory used	Dysraphism (spina bifida)	Type of hydrocephalus (number of patients)	Surgery performed (number of patients)	Imaging of suspected anomalies (number of patients)
Mali	Cefotaxime (70%)	Paracetamol (100%)	Nifluril (70%), Profenid (30%)	4	Tetraventricular (3); triventricular (8); no dilation hydrocephaly (1)	VCS (7); VCS + spinal surgery (4); VCS + biopsy (1)	Dandy-Walker syndrome (1); interventricular tumor (1)
Kinshasa	Cefotaxime (70%), ceftriaxone (30%)	Paracetamol (100%)	Nifluril (70%), Profenid (30%)	4	Tetraventricular (3); triventricular (23); no dilation hydrocephaly (3)	VCS (25); VCS + spinal surgery (4)	N/A

The treatment regimens in Mali and Kinshasa show a consistent approach in terms of medication used for pain and inflammation. However, the broader range of antibiotics used in Kinshasa may reflect a response to different bacterial profiles or resistance patterns. The types of hydrocephaly and surgical interventions indicate that triventricular hydrocephaly is more common in both groups, with surgeries predominantly being shunting procedures. The additional surgeries like spinal operations or biopsies in Mali suggest that some cases may present with more complex clinical pictures.

The presence of conditions like Dandy-Walker syndrome in the Mali group underlines the importance of comprehensive diagnostic imaging in the management of hydrocephaly, allowing for tailored surgical and medical treatment. Overall, the data imply a structured approach to managing hydrocephaly in both groups, with variations likely driven by the individual clinical needs of the patients, regional medical practices, and available resources.

## Discussion

Hydrocephalus poses considerable challenges globally, particularly in regions with limited resources and healthcare infrastructure, such as many parts of Africa. The impact of hydrocephalus in Africa is profound, affecting individuals across all age groups but particularly burdening pediatric populations [9]. One of the key challenges in managing hydrocephalus in Africa is the limited access to neurosurgical care and specialized interventions [1,2,7,10]. This lack of access is often exacerbated by factors such as geographical barriers, financial constraints, and a shortage of trained healthcare professionals, including neurosurgeons and neurologists [9]. As a result, many individuals with hydrocephalus in Africa face delays in diagnosis, inadequate treatment options, and suboptimal outcomes [4]. Furthermore, the etiology of hydrocephalus in Africa may differ from that in other regions, with conditions such as postinfectious hydrocephalus due to meningitis or encephalitis being more prevalent [10-12]. These conditions highlight the intersection of infectious diseases and neurological disorders, adding another layer of complexity to the management of hydrocephalus in this context.

The socioeconomic impact of hydrocephalus in Africa is also significant, as it often affects vulnerable populations who may already be marginalized due to factors such as poverty, limited access to education, and inadequate healthcare infrastructure [1]. Families of individuals with hydrocephalus may face financial hardships due to the costs associated with medical care, including diagnostic tests, surgical interventions, and postoperative care [13]. To address these challenges, there is a growing recognition of the need for innovative and sustainable approaches to the management of hydrocephalus in Africa [12]. EVC, including techniques such as ETV, has emerged as a promising alternative to traditional treatments like VP shunts [14]. EVC offers a minimally invasive option that can reduce the risk of complications such as infections and mechanical failures, which are particularly relevant in resource-limited settings. The role of VCS in the management of hydrocephalus is a topic of growing interest and significance, especially in regions like the city province of Kinshasa, where healthcare disparities and resource limitations are prevalent. In Mali and the Democratic Republic of the Congo, it has been estimated that the cost of surgery and hospitalization for an ETV is about 3,000 US dollars, while the cost of a VP shunt is about 2,500 US dollars. Studies conducted by Warf BC, which assess the feasibility, advantages, and outcomes of VCS compared to VP shunts, contribute valuable insights into optimizing neurosurgical care for hydrocephalus patients in Africa [15-17]. By addressing the specific challenges and tailoring interventions to the local healthcare landscape, strides can be made toward enhancing the quality of care and outcomes for individuals living with hydrocephalus in Africa [18]. However, the feasibility and effectiveness of EVC in Africa, including regions like the city province of Kinshasa in the DRC, need to be carefully evaluated. Factors such as access to specialized equipment, training of healthcare professionals, patient selection criteria, and long-term outcomes must be considered to determine the role of EVC in improving the management of hydrocephalus in this context.

Feasibility of ventriculocisternostomy in Kinshasa

The feasibility of VCS in Kinshasa's healthcare environment hinges on several factors. The availability of trained neurosurgeons proficient in endoscopic techniques is crucial. Studies have shown that the success of VCS, particularly ETV, is highly dependent on the surgeon's expertise and experience [19,20]. Therefore, investing in training programs and building local neurosurgical capacity becomes imperative, and the infrastructure and equipment required for performing VCS need to be in place. This includes high-resolution endoscopes, imaging facilities for preoperative planning and postoperative monitoring, and a well-equipped surgical suite. Collaborations with international organizations or initiatives focusing on neurosurgical capacity-building, such as the East African Neurosurgical Research Collaboration (Warf BC; East African Neurosurgical Research Collaboration, 2010), could aid in overcoming equipment-related challenges [10]. Patient selection criteria must be refined to identify suitable candidates for VCS. While ETV has shown favorable outcomes in obstructive hydrocephalus, careful patient evaluation is needed to assess the underlying pathology, anatomical considerations, and potential risks [21-23]. Multidisciplinary teams comprising neurosurgeons, neurologists, radiologists, and pediatricians can collaboratively determine the appropriateness of VCS for individual cases.

Advantages of ventriculocisternostomy over ventriculoperitoneal shunts

Comparing VCS with VP shunts reveals several advantages that make VCS a compelling option, particularly in resource-constrained settings like Kinshasa.

Reduced Infection Risk

One of the primary benefits of VCS is the reduced risk of infection compared to VP shunts. VP shunts are associated with a significant incidence of shunt-related infections, often necessitating revision surgeries and antimicrobial therapy [3]. In contrast, VCS eliminates the need for intraperitoneal foreign bodies, minimizing infection risks and long-term antibiotic usage.

Lower Mechanical Failure Rates

VP shunts are prone to mechanical failures such as obstruction, disconnection, or migration, leading to shunt malfunction and subsequent complications [2]. VCS, being a shunt-free technique, mitigates these mechanical failure risks, offering more durable and sustainable hydrocephalus management.

Minimally Invasive Approach

ETV, a form of VCS, is a minimally invasive procedure compared to the invasive nature of VP shunt placement. Minimally invasive techniques contribute to shorter hospital stays, faster recovery times, and reduced healthcare costs [9].

Long-Term Cost-Effectiveness

While the initial equipment and training costs for VCS may be higher, the long-term cost-effectiveness of avoiding shunt-related complications, revisions, and hospitalizations makes VCS a financially viable option [14].

Comparative analysis of clinical data

Implementing VCS aligns with the goals of sustainable healthcare delivery by reducing the burden of chronic conditions, optimizing resource utilization, and promoting locally driven neurosurgical expertise and infrastructure development [1]. The clinical data provided offers valuable insights into the demographic characteristics, treatment modalities, and outcomes of patients undergoing VCS in Mali and Kinshasa. The analysis reveals similarities and differences that warrant further exploration and consideration in the context of VCS feasibility and outcomes. The demographic data, including age, birth weight, hospital stay, fever during pregnancy, and consanguinity, provides a foundational understanding of the patient population. While there were no statistically significant differences in age or birth weight between Mali and Kinshasa groups, trends such as longer hospital stays in Kinshasa may reflect logistical or healthcare system variations [2,3,24,25].

Type of Hydrocephalus and Surgical Interventions

The predominance of triventricular hydrocephalus and the utilization of VCS as the primary surgical intervention aligns with established clinical patterns [2]. Additional surgeries such as spinal procedures indicate the complexity of some cases and the need for tailored surgical approaches.

The role of genetics in the etiology and progression of hydrocephalus is an emerging area of interest. Studies exploring the genetic predispositions to hydrocephalus suggest that certain populations may have varying risks and outcomes associated with the condition [24-26]. In Kinshasa, where consanguinity rates are notable, genetic counseling and screening could play a significant role in the early detection and management of hydrocephalus, potentially influencing the selection criteria for VCS [27].

The incidence of postoperative fever and mortality rates within 30 days highlights the importance of postoperative care protocols and ongoing surveillance. The comparable rates between the Mali and Kinshasa groups suggest standardized clinical management despite contextual differences. The consistent use of antibiotics, analgesics, and anti-inflammatory medications across both groups underscores standardized clinical protocols. However, variations in the antibiotic choice between cefotaxime and ceftriaxone suggest potential regional differences in microbial profiles or prescribing practices.

Limitations and future directions

While the data provide valuable insights, several limitations must be acknowledged. The study could be expanded to include long-term follow-up of patients beyond the immediate postoperative period, aiming to assess the durability of VCS outcomes, the incidence of late complications, and the need for reoperation. Additionally, evaluating patients' quality of life post-surgery could provide insights into the functional and social impacts of this treatment modality, offering a more holistic view of its benefits.

Comparative Effectiveness Research

While the study mentions the advantages of VCS over traditional shunting techniques, future research could explicitly include a comparative effectiveness research (CER) component. This would involve directly comparing the outcomes of VCS with those of VP shunts in a controlled setting, accounting for variables such as infection rates, mechanical failure, and overall survival rates. Such comparative studies could further solidify the evidence base supporting VCS' role in managing hydrocephalus.

Socioeconomic Impact Analysis

The socioeconomic implications of hydrocephalus and its management strategies are profound, especially in resource-limited settings like the DRC. Future expansions of this study could incorporate a socioeconomic impact analysis, evaluating the financial burden on families and healthcare systems associated with VCS versus shunt surgeries. This analysis could include direct medical costs, indirect costs, such as lost caregiver wages, and the impact of improved neurosurgical care accessibility on these economic factors.

Integration of Advanced Diagnostic and Monitoring Technologies

The study highlights the utilization of state-of-the-art endoscopic equipment for VCS. Future studies could explore the integration of advanced diagnostic and monitoring technologies, such as intraoperative neuroimaging, telesurgery capabilities for remote training and assistance, and postoperative telemonitoring for early detection of complications. These technologies could enhance surgical precision, training outreach, and postoperative care, especially in remote or underserved areas.

Genetic and Environmental Factors

Considering the potential genetic predispositions to hydrocephalus noted in the study, future research could delve deeper into the genetic and environmental factors contributing to hydrocephalus in the DRC and other African regions. This could involve genetic screening and environmental assessments to identify risk factors, which, in turn, could inform targeted prevention and early intervention strategies.

Implementation Science

Finally, expanding the study to include elements of implementation science could provide valuable insights into the barriers and facilitators affecting the adoption of VCS in resource-limited settings. This could help identify effective strategies for training, equipment procurement, and procedural standardization, facilitating the broader implementation of VCS across the DRC and similar contexts.

## Conclusions

VCS, especially ETV, emerges as a formidable alternative for hydrocephalus management in Mali and DRC, showcasing the potential to markedly ameliorate patient outcomes, economize healthcare expenditures, and fortify the local neurosurgical capacity. The fruition of ETV's full potential in Kinshasa is contingent upon a collaborative strategy that emphasizes training, infrastructural enhancements, and a comprehensive patient care ethos, all while steadfastly addressing the broader socioeconomic impediments that influence health outcomes within the region.
